# Molecular Insight into the Therapeutic Promise of Targeting *APOE4* for Alzheimer's Disease

**DOI:** 10.1155/2020/5086250

**Published:** 2020-05-15

**Authors:** Abdullah Al Mamun, Md. Sahab Uddin, Md. Fahim Bin Bashar, Sonia Zaman, Yesmin Begum, Israt Jahan Bulbul, Md. Siddiqul Islam, Md. Shahid Sarwar, Bijo Mathew, Md. Shah Amran, Ghulam Md Ashraf, May N. Bin-Jumah, Shaker A. Mousa, Mohamed M. Abdel-Daim

**Affiliations:** ^1^Department of Pharmacy, Southeast University, Dhaka, Bangladesh; ^2^Pharmakon Neuroscience Research Network, Dhaka, Bangladesh; ^3^Department of Pharmacy, University of Development Alternative, Dhaka, Bangladesh; ^4^Department of Pharmacy, Noakhali Science and Technology University, Noakhali, Bangladesh; ^5^Division of Drug Design and Medicinal Chemistry Research Lab, Department of Pharmaceutical Chemistry, Ahalia School of Pharmacy, Palakkad, India; ^6^Department of Pharmaceutical Chemistry, University of Dhaka, Dhaka, Bangladesh; ^7^King Fahd Medical Research Center, King Abdulaziz University, Jeddah, Saudi Arabia; ^8^Department of Medical Laboratory Technology, Faculty of Applied Medical Sciences, King Abdulaziz University, Jeddah, Saudi Arabia; ^9^Department of Biology, College of Science, Princess Nourah bint Abdulrahman University, Riyadh 11474, Saudi Arabia; ^10^Pharmaceutical Research Institute, Albany College of Pharmacy and Health Sciences, New York, NY 12144, USA; ^11^Department of Zoology, College of Science, King Saud University, P.O. Box 2455, Riyadh 11451, Saudi Arabia; ^12^Pharmacology Department, Faculty of Veterinary Medicine, Suez Canal University, Ismailia 41522, Egypt

## Abstract

Alzheimer's disease (AD) is a progressive neurodegenerative disease that causes chronic cognitive dysfunction. Most of the AD cases are late onset, and the apolipoprotein E (*APOE*) isoform is a key genetic risk factor. The *APOE* gene has 3 key alleles in humans including *APOE2*, *APOE3*, and *APOE4*. Among them, *APOE4* is the most potent genetic risk factor for late-onset AD (LOAD), while *APOE2* has a defensive effect. Research data suggest that *APOE4* leads to the pathogenesis of AD through various processes such as accelerated beta-amyloid aggregations that raised neurofibrillary tangle formation, cerebrovascular diseases, aggravated neuroinflammation, and synaptic loss. However, the precise mode of actions regarding in what way *APOE4* leads to AD pathology remains unclear. Since *APOE* contributes to several pathological pathways of AD, targeting *APOE4* might serve as a promising strategy for the development of novel drugs to combat AD. In this review, we focus on the recent studies about *APOE4*-targeted therapeutic strategies that have been advanced in animal models and are being prepared for use in humans for the management of AD.

## 1. Introduction

Alzheimer's disease (AD) is a neurodegenerative disorder in which the death of nerve cells causes memory loss and cognitive decline that is serious enough to interfere with daily life [1–4]. Neuropathologically, AD is characterized by the deposition of extracellular beta-amyloid (A*β*) as well as increased intracellular neurofibrillary tangles along with the activation of glia and neuronal death [5–9]. Besides environmental and lifestyle-related risk factors, genetic constituents are also deliberated to raise the risk of progressing AD [10, 11]. Undeniably, research has detected several loci connected with AD, such as both pathogenic and susceptibility genes. AD is categorized into 2 forms according to the age of onset, including early-onset AD (EOAD) that begins in people who are below 65 years old and it is estimated to be about 1–5% of all cases, and late-onset AD (LOAD) that starts in people aged 65 or above 65 and it is projected to be greater than 95% of all cases [12]. There are 3 leading congenital gene mutations, such as presenilin-1 (*PSEN1*), presenilin-2 (*PSEN2*), and amyloid precursor protein (APP), which play a central role in raising the generation of A*β* and have been directly related to EOAD [13–15]. On the contrary, LOAD has been detected to be more complicated and is linked to several genes with increased vulnerability [16]. Genetically, the apolipoprotein E4 (*APOE4*) allele is considered as the paramount determinant for LOAD [17–20].

People who inherit one copy of the *APOE4* allele may cause a higher risk of raising AD, and people who possess two copies of the *APOE4* allele are at greater risk of progressing AD [21, 22]. The *APOE4* gene may also be accompanied by an earlier onset of memory dysfunction and other symptoms in comparison with AD patients who do not have this gene. It is unknown how the *APOE4* allele is connected with the risk of AD. Conversely, the *APOE4* gene is also linked with a greater number of protein clusters called amyloid plaques, which are found in the AD brain tissue [23, 24]. Furthermore, an aggregation of amyloid plaques is greatly responsible for the death of neurons and the developing symptoms of AD [25, 26]. It has been found that the interaction between herpes simplex virus type 1 (HSV-1) and *APOE* isoforms indicate a connection between HSV-1 deoxyribonucleic acid (DNA) detection in AD tissues and the existence of the *APOE4* allele [27, 28]. In addition, recent investigations have shown a potential relationship between *APOE* isoform-dependent modifications in tau pathology and neurodegeneration [29]. Therefore, it is evident that people who have the *APOE4* allele inherit a greater risk of progressing AD, not the disease itself [30].

Oxidative stress has also been connected with APOE4 in AD patients. ApoE4 is linked to higher oxidative stress as well as diminished antioxidant enzyme activity in the hippocampus of AD patients [31–33]. Oxidative stress markers such as increased oxidized proteins, glycosylated products, elevated levels of lipid peroxidation, formation of aldehydes, alcohols, ketones, free carbonyls, and cholestenone, as well as oxidative modifications in ribonucleic acid (RNA) and mitochondrial and nuclear DNA were observed in postmortem brain tissue and in peripheral systems such as cells and isolated mitochondria from initial phases of AD and *APOE4* carriers [34–51]. On the other hand, APOE was shown to act as an antioxidant directly or indirectly against hydrogen peroxide-mediated cytotoxicity in a B12 APOE expressing cell line [52]. According to the study by Hayek et al. [53], the increased levels of peroxidized plasma low-density lipoproteins in APOE-deficient mice were observed. Furthermore, the levels of lipid oxidation were considerably elevated in the frontal cortex of AD patients who were heterozygous or homozygous *APOE4* carriers compared to homozygous *APOE3* carriers and controls [54]. Although upregulation of catalase activity was completely found in the frontal cortex tissue of homozygous *APOE4* carriers, the activities of superoxide dismutase and glutathione concentrations were not as different as those from controls [54].

Hitherto, there are no approved drugs directly targeting APOE4, even though *APOE4* was detected about 25 years ago [55–57]. Hence, due to its genetic predominance, *APOE* isoforms have turned into an auspicious target for better understanding the pathophysiological pathways of AD, identifying patients who are at greater risk for the progression of AD and opening a novel therapeutic approach against AD. Moreover, some clinical researches both in animals and humans have verified that *APOE4* remarkably affects the various independent biological pathways in the brain which play a pivotal role in the development of AD [55, 58]. In this review, we emphasize the current studies regarding *APOE4*-targeted promising therapeutic strategies to combat AD pathogenesis.

## 2. *APOE*—Polymorphism and Susceptibility to Alzheimer's Disease

**Figure 1:**
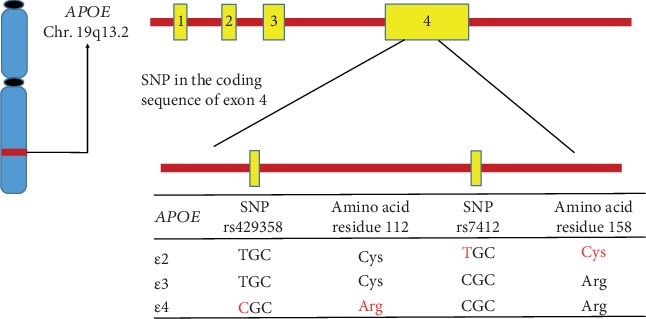
Schematic presentation of the human APOE genotype and APOE polymorphisms. The human APOE gene is situated in the long arm of chromosome 19. In exon 4 of chromosome 19, two nonsynonymous single nucleotide polymorphisms (SNPs) including rs429358 and rs7412 produce 3 main allelic variants (E2, E3, and E4). The resulting *APOE2*, *APOE3*, and *APOE4* isoforms vary from one another at amino acid residues 112 or 158.

APOE is a chylomicron lipoprotein that is necessary for the transport of lipids and the metabolism of lipoproteins, and it is encoded by the *APOE* gene which is situated in the long arm of chromosome 19q13.2 [59, 60] (Figure 1). The *APOE* gene has 3 variants, called *APOE2*, *APOE3*, and *APOE4*, which are present at ~7%, 79%, and ~14%, respectively in the whole populace, and these show dissimilarities in lipid- as well as receptor-binding efficiency. The *APOE* allelic proteins vary by merely 1 or 2 amino acids including cysteine and arginine at residues 112 and 158, with *APOE2* (cys112, cys158), *APOE3* (cys112, arg158), and *APOE4* (arg112, arg158) [61, 62]. Although the *APOE2* gene is associated with type III hyperlipoproteinemia, however, it has a defensive effect against AD. The *APOE3* is the most predominant allele and doesn't seem to influence risk. Conversely, the *APOE4* gene is connected with a greater risk for AD and coronary artery disease [63, 64]. The morphology of the APOE4 protein reduces the capability of *APOE4* to remove the A*β* protein from the brain, causing the development of AD. The existence of one copy of the *APOE4* allele raises the AD risk by 3 times, whereas people who inherit two copies of *APOE4* alleles are 8 times more likely to progress with AD in comparison with those lacking any *APOE4* allele [56]. These data recommend that *APOE4* is the highest identified genetic risk factor for AD compared to any other genes so far.

## 3. APOE-Targeted Treatment for Alzheimer's Disease

**Figure 2:**
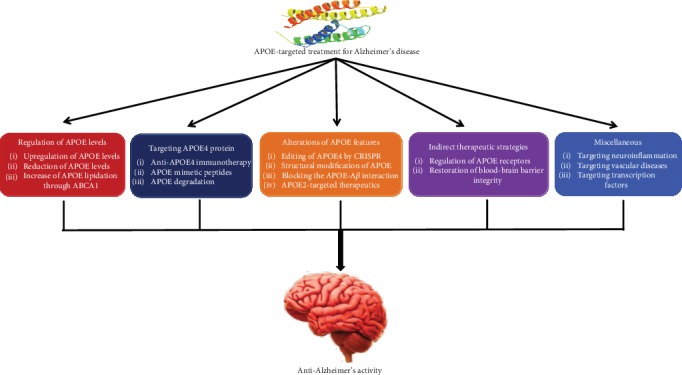
Classification of APOE-targeted treatment strategies for Alzheimer's disease.

It has been suggested that the *APOE4* allele considerably alters various biological pathways toward vulnerable conditions for the progression of AD. It is evident that *APOE* modifies multiple biological pathways by its analogous protein APOE, whereas the key mechanism connecting with *APOE4* and neurodegeneration until now remains unclear. Hence, alterations in the *APOE* allele, along with APOE structures, are fortunate targets for novel drug design and treatment for AD. In this appraisal, we emphasize on the aspects of APOE4 for which therapeutic strategies are being developed (Figure 2). There are several comprehensive studies demonstrating the molecular mechanisms underlying the effects of APOE4 [65–69]. Furthermore, some therapeutic strategies for targeting APOE in AD [70] are being undertaken in the scientific community (Table 1). We emphasized the diverse characteristics of APOE4 via which therapeutic strategies are advanced, and these are discussed below.

### 3.1. Regulation of APOE Levels

#### 3.1.1. Upregulation of APOE Levels

Several studies for the hallmarks of AD have revealed contradictory outcomes regarding whether the quantity of APOE in plasma and CSF are decreased in AD patients in comparison with healthy individuals [71–74]. However, many animal studies demonstrated the therapeutic potential of compounds that raise APOE levels in the brain [75–91]. *APOE* transcription is positively controlled by retinoid X receptors (RXRs) and nuclear receptors, as well as liver X receptors (LXRs) which generate heterodimers [92]. Actually, the oral intake of an RXR agonist including bexarotene raised the levels of APOE in the brain, decreased the accumulation of A*β*, and enhanced cognitive abilities in the amyloid mice model [76]. Furthermore, bexarotene has already been recognized as a drug by the Food and Drug Administration (FDA) for the treatment of cutaneous T-cell lymphoma [93–96], and the emerging application of this drug for the treatment of AD has been receiving the attention of numerous researchers for many years. Clinical research recommended that bexarotene considerably reduced cognitive dysfunction in the amyloid model mouse, which expresses human *APOE3* and *APOE4* [77], as well as returned the *APOE4*-mediated neuronal and cognitive dysfunctions in mice devoid of amyloid background [75]. In addition, bexarotene is also useful for the restoration of age-dependent synaptic proteins' loss [97]. Conversely, there are contradictory data about the negative effects of bexarotene therapy on amyloid pathology in animal studies [77, 79, 81, 82, 97]. Additionally, bexarotene showed adverse effects such as hepatic failure in mice models [78, 80]. A study by Cummings et al. [98] demonstrated that 4 weeks of bexarotene therapy in the AD human model did not diminish amyloid plaque in the brain as determined by positron emission tomography scans, although the size of the sample was noticeably small. Hence, although an RXR agonist including bexarotene has a promising effect on stopping the pathogenesis of AD, further evaluations as well as dosage optimization are necessary for its emerging therapeutic use for the treatment of AD.

#### 3.1.2. Reduction of APOE Levels

Current studies have claimed that *APOE* haploinsufficiency in APOE-targeted-replacement (TR) mice weakens the accumulation of A*β* despite *APOE* genotypes [99, 100]. It is evident that the intraperitoneal injection of an anti-APOE antibody has also been exposed to enhance spatial learning ability by decreasing the quantity of soluble APOE in the brain and slowing the accumulation of A*β* in the brain in an amyloid animal model with no obvious side effects [101, 102]. Although the reduction of APOE levels may be useful for ameliorating the pathology of A*β*; however, this strategy must be justified cautiously for clinical use because the lack of APOE can cause severe hyperlipidemia as reported to take place in an APOE-lacking individual homozygous for an ablative APOE frameshift mutation [103].

*APOE4* is widely accepted as an isoform that leads to the pathogenesis of AD by not only gain-of-functional but also loss-of-functional characteristics in comparison with *APOE3* [19, 104]. Therefore, these bidirectional properties of *APOE4* are necessary to be deliberated upon for advancing favorable APOE-targeted treatments for AD. Furthermore, targeted removal of APOE4 will be a hopeful approach to lessen its toxicity. A study by Luz et al. [105] showed that anti-APOE4 monoclonal antibody (9D11) therapy might prevent the APOE4-induced cognitive damage and the hyperphosphorylation of tau in the brain in APOE4 mice, even though follow-up investigations are mandatory. Moreover, some inactive types of APOE such as APOE fragments and depositions are expected to be detrimental [106–110] where APOE4 possibly has a greater chance of adopting such structural alterations or proteolysis than other polymorphisms [111]. Hence, eliminating APOE depositions but not the original form of functional APOE with the help of immunodepletion can be a different therapeutic approach. Pharmacological strategies to remove APOE fragmentation and depositions might also be considered as potential therapeutic approaches.

#### 3.1.3. Increase of APOE Lipidation through ABCA1

**Figure 3:**
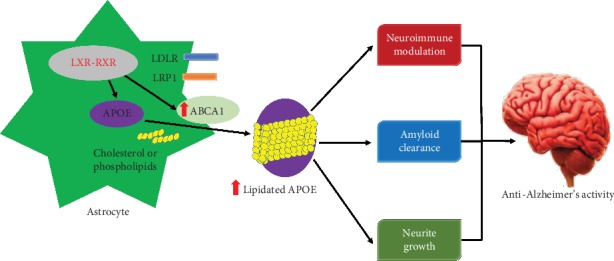
Increasing lipidation of the astrocytic pool of APOE. APOE is principally produced by astrocytes in the brain. Activation of nuclear hormone receptors including LXR and RXR plays an essential role in the expression of APOE and ABCA1. Moreover, ABCA1 is very important for the lipidation of APOE and increasing the expression of ABCA1 contributes to enhanced lipidation of all APOE polymorphisms (indicated by red arrows). It is assumed that enhanced lipidation of APOE will be valuable for several AD-relevant endpoints.

The lipidation condition of APOE considerably affects its activity [112], since the APOE protein (biologically active) is connected with lipids [113, 114]. ATP-binding cassette subfamily A member 1 (ABCA1) is responsible for loading lipid particles on APOE proteins in the brain [115]. The removal of ABCA1 reduces the production of suitably lipidated APOE particles leading to the highly amyloidogenic pathologies in an amyloid mice model [116]. Conversely, *ABCA1* overexpression diminishes the accumulation of A*β* in an APOE-dependent way in the amyloid mouse model [117], which recommends a favorable role of *ABCA1*-induced APOE lipidation for the stopping of the AD pathogenesis (Figure 3). As depicted, those agonists for RXRs and LXRs could lessen the accumulation of A*β* and improve cognition in an amyloid mice model and these processes regulate the transcription of *APOE* and *ABCA1* [77, 84, 91]. Furthermore, lipid transport to cells takes place as APOE is endocytosed through members of the low-density lipoprotein receptor (LDLR) family [118]; endocytosis enhances neurite outgrowth [119], synapse formation [120], and neuronal sprouting [121]. Additionally, recent investigations have found that lipidated APOE plays a pivotal role in demonstrating various functions, such as lipid/cholesterol transport, regeneration of synapse, immune modulation, and clearance of A*β* [85, 102, 122, 123]. Therefore, these examinations advocate the idea that raising the lipidation of APOE may be a useful strategy for the treatment of AD, even though it might be arduous to control the lipidated condition without changing APOE levels.

Furthermore, APOE4 is less lipidated than APOE2 and APOE3 both in APOE-TR mice [124, 125] and in humans [126]. Mounting evidence recommends that lipid-driven pathways may contribute to the harmful characteristics of APOE4 [127]. A study by Boehm-Cagan and Michaelson [75] demonstrated that RXR agonists including bexarotene were used in the treatment of APOE4-TR mice resulting in reversed APOE4-mediated neuronal and cognitive deficits apparently by raising APOE4 lipidation. Hence, altering the lipidation condition of APOE4 appears to be a potential therapeutic strategy [128], although the LXR/RXRs-ABCA1 axis activation can possibly raise the levels of APOE4, thus increasing the gain-of-toxic functions of APOE4 [19].

### 3.2. Targeting APOE4 Protein

#### 3.2.1. Anti-APOE4 Immunotherapy

The fundamental principle of APOE4 immunotherapy is close to that applied in the immunotherapy of tau and A*β* [129], specifically to produce or introduce antibodies against these molecules in the periphery that can neutralize their target (this strategy presumes a toxic effect of APOE4) after their penetration into the brain. In theory, the use of immunotherapy to APOE is encountered by the difficulty that the APOE level in the periphery is about 10-times greater than that in the brain [130], as a result, anti-APOE antibodies must be titrated out in the periphery prior to reaching the brain. Many studies demonstrated that the peripheral use of anti-mouse APOE antibodies in APP transgenic (TR) mice can suppress the amyloid deposition before the beginning of plaque and reduce its deposition after the formation of plaque [101, 102]. Despite the mode of actions involving these pivotal effects of the anti-APOE monoclonal antibodies, these outcomes have an enormous significance and offer a fundamental idea about the reliability of anti-APOE4 immunotherapy as a promising therapeutic strategy. Furthermore, this strategy has now been expanded to APOE3- and APOE4-directed mice using an antibody that reacts particularly with APOE4 [131]. This reveals that repetitive intraperitoneal injection of these antibodies in mice leads to their aggregation in the brain and also in the generation of APOE/IgG complexes, especially in APOE4 mice. Moreover, this was connected with the restoration of cognitive damages in APOE4 mice as well as with the restoration of central synaptic and AD-associated pathological effects of APOE4 [131].

#### 3.2.2. APOE Mimetic Peptides

Using of APOE mimetic peptides represent a further therapeutic strategy against AD. Furthermore, these tiny peptides, which either conform to the receptor-binding APOE domain [132–134] or to a discrete domain of APOE including amphipathic helix domains [134], significantly lessen the neurodegeneration after brain insults [133, 135–138] and defend against tau- and A*β*-mediated pathogenesis in TR mice and analogous model domains [132–134]. The fundamental mechanism of the defensive effects of these tiny peptides might be owing to their anti-inflammatory effects. Conversely, a study of Vitek et al. [133] revealed that these APOE mimetic peptides were defensive after brain insults in both APOE3 and APOE4 mice.

#### 3.2.3. APOE Degradation

APOE4 generates an intermediate molten globule structure, which makes it less stable when compared with APOE3 and its C- and N-terminal interaction as described earlier. Furthermore, this domain interaction in neurons makes APOE4 particularly vulnerable to discrete proteases and responsible for the production of C-terminal neurotoxic portions of APOE4 [67, 139–141]. As stress raises the neuronal APOE generation, it has been anticipated that the raised generation of intraneuronal APOE4 portions under stressful states may play a pivotal role in inducing the pathogenic actions of APOE4 [67, 139–141]. Hence, the neuronal degradation of APOE4 leads to the detection of the proteases as well as the advancement of inhibitors against them demonstrating an additional strategy for neutralizing the actions of APOE4.

### 3.3. Alterations of APOE Features

#### 3.3.1. Editing of *APOE4* by CRISPR

**Figure 4:**
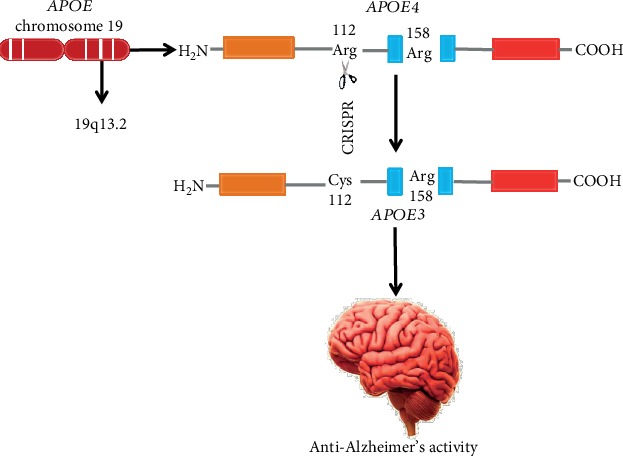
Editing of *APOE4* by CRISPR to generate an *APOE3*-like structure. The *APOE4* allele is generally produced by *APOE* chromosome 19 (19q13.2). The coding of DNA for *APOE4* varies from *APOE3*, which is considered as the benign gene for AD, by only 1 nucleotide (explicitly, the 112th position is cysteine in *APOE3* and arginine in the case of *APOE4*). Particularly, the CRISPR method could be used for converting the *APOE4* gene into *APOE3*.

The transformation of the *APOE4* gene to either *APOE2* or *APOE3* and the abolition of the concentration dissimilarity between them would play an important role in solving the crux of the APOE4 problem in spite of the poor understanding of the mechanisms regarding the effects of APOE4. It was quite impossible to enable the exact gene editing before the advancement of the clustered regularly interspaced short palindromic repeats (CRISPR) gene-editing method [142]. Furthermore, this method is specifically the opposite for the *APOE* gene, in which the coding of DNA for *APOE4* varies from that of *APOE3*, which is considered as the benign gene for AD, by merely 1 nucleotide (explicitly, the 112th position is cysteine in *APOE3* and arginine in the case of *APOE4*). Particularly, the CRISPR method can be used for transforming the *APOE4* gene into *APOE3* as given in Figure 4. Conversely, it can also be applied in an *APOE4*-knockout model where *APOE3* homozygote mice is transformed by *APOE3/APOE4* heterozygote mice, which would be predicted to be defensive when a noxious effect of APOE4 is supposed. Moreover, in a study of Komor et al. [143], CRISPR cell culture research demonstrated the precise transformation of *APOE4* to an *APOE3* derivative, and this method was also used to silence *APOE4* devoid of influencing the expression of APOE3 [144]. The later strategy might therapeutically work against the assumed toxicity related to APOE4. In contrast, effective *in vivo* use of CRISPR to APOE4 mice has not been established so far. Moreover, it is very essential to remember that the CRISPR method is in its initial stages and data are still promising about probable off-target gene modification and mosaicism, where not all copies of the target gene are edited.

#### 3.3.2. Structural Modification of APOE

**Figure 5:**
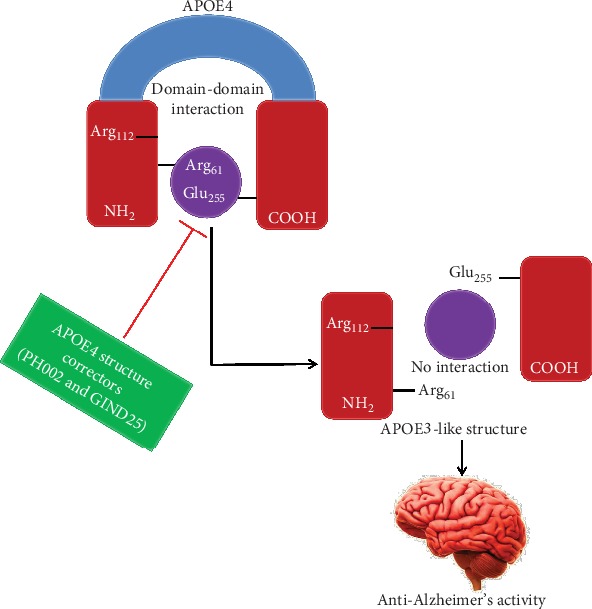
APOE4 structure correctors can disrupt ApoE4 domain-domain interaction. In the N-terminal domain, Arg-61 interacts with Glu-255 in the C-terminal domain in APOE4. APOE4 structure correctors (PH002 and GIND25) that are expected to interact with APOE4 in the region of Arg-61 would interrupt domain-domain interaction and transform APOE4 to an APOE3-like molecule.

In the N-terminal, the amino acid residues 112 and 158 are the markers of APOE polymorphisms; however, the interaction between residues Glu255 and Arg61 is possibly a further characteristic, which structurally differentiates APOE4 from APOE3. Furthermore, this domain-domain interaction probably produces an aberrant structure in APOE4 that leads to neurotoxicity [145], even though the structure of the complete, native APOE has not been examined until now. In neuronal N2A cells, the expression of APOE4 triggers mitochondrial dysfunction, although this effect is abolished in an APOE4 mutant lacking the interaction of domain (APOE4-R61T) [146]. Moreover, various molecular compounds (PH002 and GIND25) have been accounted to counter harmful actions of APOE4 by obstructing the domain-domain interaction [147] as shown in Figure 5. Hence, altering the pathological conformation of APOE4 including the domain-domain interaction in neurons might be a potential therapeutic strategy for the treatment of AD. Conversely, the effectiveness of these compounds on AD-linked processes has not been determined *in vivo* so far.

On the other hand, AD might be influenced both by the sequence of DNA and by epigenetic profiles in the *APOE* region. Remarkably, epigenetic mechanisms, including DNA methylation, histone modification, and noncoding RNA can control the expression of the gene whereas the fundamental sequence of DNA remains the same. The epigenome is also influenced both by basic genetic variants and by environmental factors such as environmental pollutants, social environment, and health behaviors [148]. Methylation of cytosine-phosphate-guanine (CpG) dinucleotides is the best understood epigenetic mechanism. It has been recognized that epigenomic patterns of DNA methylation alter with age [149]. In a study by Ma et al. [150], reported that methylation levels at different CpG sites in *APOE* were considerably connected with age in lymphocytes, and these age-related alterations in DNA methylation were altered by *APOE* genetic variants. Thus, epigenetic regulation can be a strategy to combat AD pathogenesis.

#### 3.3.3. Blocking the APOE-A*β* Interaction

Although the interaction of APOE with A*β* might be negligible under physiological states [151], A*β* and APOE colocalize in amyloid plaques in AD brains [152]. It is widely accepted that the interaction between A*β* and APOE induces the accumulation of A*β* in human brains [5]. Undeniably, suppression of the interaction of APOE with A*β* by an artificial peptide (A*β* 12-28P, analogous to the APOE binding site on the complete A*β*) played a crucial role in reducing the aggregation of A*β* and intraneuronal deposition of A*β*, and improved memory dysfunctions in amyloid animal models [153, 154]. According to the study of Liu et al. [155] on the 3xTg AD mouse model, tau and A*β* pathology were both sheltered and the interaction of APOE with A*β* was obstructed by A*β* 12-28P, resulting in the diminished accumulation of A*β* and the aggregation of insoluble tau in AD brain. Moreover, the therapy with A*β* 12-28P played an essential role in decreasing the oligomers of A*β* and a load of amyloid plaque also improved neuritic deterioration in the amyloid model mouse with an APOE4-TR or APOE2-TR mouse background [156]. Therefore, suppression of the interaction between A*β* and APOE seems to be favorable for stopping the accumulation of A*β* despite the APOE polymorphisms, although the pharmacological effect of artificial peptides may vary according to the APOE polymorphism being targeted. Additionally, Hao et al. [157] demonstrated that A*β* 20–29 peptides might obstruct the interaction of APOE with A*β*, thus decreasing the fibrillogenesis as well as cytotoxicity of A*βin vitro*. Captivatingly, immunotherapy of APOE noticeably reduces the accumulation of A*β* in amyloid mouse models as discussed earlier [101, 102]. Recently, in an *in vivo* test, using antibodies that accept both human APOE3 and APOE4 and then combining specifically to nonlipidated APOE rather than lipidated APOE result in diminishing the accumulation of A*β* in a TR mice model [158]. In addition, APOE antisense oligonucleotides are also used for the lessening of amyloid plaques [159]. It is evident that APOE3 and APOE4 bind diversely and directly to A*β* [125]; however, a study by Verghese et al. [151] revealed that the interaction of APOE4 with A*β* might be indirect as well as intervened through a third molecule. Therefore, blocking the interaction between APOE4 and A*β* may be a promising therapeutic strategy for the treatment of AD.

#### 3.3.4. APOE2-Targeted Therapeutics

The occurrence of APOE2 in AD patients is about 2-times lower than the general populace, and it is connected with less noticeable brain pathology than that found in non-APOE2 AD subjects [160]. The heterozygosity of *APOE2* is also linked with longevity [160] and lessened age-dependent cognitive dysfunction [161–164]. In addition, APOE2 increases the neuroprotective activity against AD via various molecular mechanisms [165]. Consequently, in the case of neurodegenerative disorders that are directly linked with neuronal and synaptic loss, APOE2 is defensive because of its capability to excite the restoration of these pathways. Various investigations recommended that the brain pathological effects of APOE4 in TR mice could be counteracted by the injection (intracerebral) of viral vectors expressing APOE2 [166, 167], recommending a new anti-APOE4 therapeutic strategy [168]. According to the study of Hu et al. [169], APOE4 is hypolipidated compared with APOE3 and APOE2 is hyperlipidated in relation to APOE3. Moreover, it is evident that APOE2 and APOE4 affect the identical pathway such as lipidation of APOE; however, they drive in opposite directions. In contrast, the probability that APOE4 and APOE2 activate through diverse nonoverlapping processes with contrasting physiological outcomes could not be denied.

### 3.4. Indirect Therapeutic Strategies

#### 3.4.1. Regulation of APOE Receptors

The LDLR family, including LDLR and low-density lipoprotein receptor-related protein 1 (LRP1) [170], plays a central role in mediating the endocytosis of APOE. APOE accelerates the cellular uptake of A*β* via these receptors either by producing the complexes of APOE/A*β* or by inhibiting the A*β* interaction through competition for the binding receptor. Furthermore, diverse mechanisms including APOE isoform-dependent, lipidation-status-dependent, and concentration-dependent mechanisms [112, 170] are responsible for complicating these events. Captivatingly, aggregating data have demonstrated the crucial roles of LDLR as well as LRP1 in the clearance of A*β* in the brain and the metabolism of lipids [171–174]; however, how APOE mediated these phenotypes remains unclear. Therefore, raising LDLR and LRP1 can be considered as a therapeutic strategy to trigger the clearance processes of A*β* in human AD. Undeniably, some compounds such as fluvastatin, caffeine, and rifampicin probably have defensive actions for the management of AD by mounting levels of LRP1 [175, 176]. Moreover, LRP1 and a different APOE receptor including APOE receptor 2 (APOER2) are playing key roles in controlling synaptic activities [170, 177]. On the other hand, a large extracellular matrix glycoprotein, for example, reelin, improves the activity of synaptic glutamate receptors via APOER2, and APOE4 interrupts this process by damaging the recycling process of APOER2 [178]. A study by Gilat-Frenkel et al. [69] showed that the levels of APOER2 in the brain hippocampus are also decreased particularly in APOE4-TR mice. Hence, controlling the levels of APOER2 can serve as an APOE-driven anti-AD treatment, even though additional researches are required.

#### 3.4.2. Restoration of Blood-Brain Barrier Integrity

According to the study of Bell et al. [179], the expression of APOE4 in astrocytes activated the proinflammatory cyclophilin A- (CyA-) nuclear factor *κ*B-matrix metalloproteinase 9 (MMP-9) processes in brain pericytes, thus resulting in the degradation of the tight junction protein and the breakdown of the blood-brain barrier (BBB) in an animal model. Prominently, these pathologies of BBB in APOE4-TR mice were restored by the treatment of cyclosporine A [179]. Age-dependent BBB breakdown as determined by the QAlb index along with raised CyA and active levels of MMP-9 were identified in CSF from cognitively regular *APOE4* carriers [179]. Therefore, cyclosporine A may be considered as a therapeutic agent for the treatment of AD, whereas it is still debatable whether APOE4-driven BBB breakdown is adequate to change the worldwide homeostatic capability of BBB leading to the pathogenesis of AD [180, 181].

### 3.5. Miscellaneous

#### 3.5.1. Targeting Neuroinflammation

Various inflammation-related targets have been recommended, such as microglia, where the current detection of gene expression patterns connected with diverse phases of the activation of microglia presents new targets wherein microglial activation can be controlled [182, 183] and which have been demonstrated to be useful in neurodegeneration-linked models [184, 185]. Furthermore, these advancements and the relationship of APOE4 with raised neuroinflammation recommend that inflammation-linked therapies can be specifically favorable in *APOE4* carriers. Conversely, neuroinflammation is a double-edged sword, supposed to be defensive at initial phases and pathological at consequent chronic phases. The use of APOE4- and AD-linked immunotherapeutic approaches is thereby anticipated to be dependent on the inflammatory reaction by which AD patients are treated. In addition, this may differ in diverse brain regions. Therefore, novel hallmarks that detect the phase and brain area of neuroinflammation are required to solve this matter.

#### 3.5.2. Targeting Vascular Diseases

There are some vascular risk factors including atherosclerosis, diabetes, and hypertension that raise the AD risk [186, 187]. Furthermore, APOE4 is connected with the raised risk for atherosclerosis and vascular dementia [188, 189] as well as with damaged vasculature integrity and BBB [189]; therefore, it is suggested that the role of APOE4 in the pathogenesis of AD may be mediated partly by a vascular constituent. Hence, the detection of the molecules by which the AD-linked vascular effects of APOE4 are driven, and which can, therefore, be considered as an AD-APOE4 vascular therapeutic approach, remains presently unclear [186]. Conversely, as significant features of vascular diseases could be treated pharmacologically and by lifestyle changes [190], such strategies are anticipated to lessen the role of vascular and APOE4/vascular pathology to AD.

#### 3.5.3. Targeting Transcription Factors

Generally, the pathological mechanisms of APOE4 are mediated extracellularly or through membrane transport and cytosolic pathways, but it has currently been recommended that APOE4 also goes through nuclear translocation and it combines particularly with high affinity to copious DNA sites [191]. Most of these sites are located in promoter areas, recommending that APOE4 can work as a transcription factor for a lot of diverse genes such as autophagy and growth factor-related genes [192, 193]. A study by Rohn [194] demonstrated that APOE4 is contained in the nucleus and this pathway is associated with a particular proteolytic APOE4 degradation. Furthermore, these investigations and outcomes suggested that APOE4 combines with the genes' promoters involved in diverse pathways that are linked with aging and AD [195] contributing to the exciting recommendation that APOE4 could serve as a transcription factor. There are many queries that remain to be investigated, including in what way APOE escapes the endoplasmic reticulum and is transferred to the nucleus as well as what is the influence of this mechanism compared with other pathological pathways.

## 4. Conclusions

In this review, we discuss several *APOE4*-mediated strategies, varying from the *APOE* gene to the APOE protein and its interacting molecules not only in cellular but also in animal model systems. These investigational strategies have been advanced to act against the pathological effects of *APOE4* in the animal model. The role of *APOE4* in raising AD risk is multifaceted, linking a wide range of cell types and activities, which needs to be considered for *APOE*-mediated drug development. Currently, there is no established proof for the human *APOE4*-mediated therapeutic investigations and it is expected that developments in animal studies will offer auspicious opportunity for the advancement of novel *APOE*-targeted treatment for AD.

## Figures and Tables

**Table 1:** Outline of therapeutic agents for targeting APOE4 for Alzheimer's disease.

Therapeutic strategies	Principle	Studies		References
		Animal models	Humans	
RXR, LXR, PPAR*γ* agonists	Increases the lipidation of APOE and promotes A*β* clearance	Mouse	Yes	[75–90, 91, 98]
Anti-APOE4 monoclonal antibody	Increases amyloid clearance and decreases APOE-associated toxic effects		Yes	[99–102, 122, 196]
Small peptides comprising the receptor-binding region in APOE	Reduces inflammation and neurotoxicity, increases APOE3-linked protective functions	Mouse	No	[123, 132, 133, 197, 198]
Small molecules	Increases A*β* clearance, APOE signaling, and cholesterol transport	Mouse	No	[175, 176]
APOE4 structure correctors (GIND25 and PH002)	Interferes with domain-domain interaction in APOE4 thus reducing its toxic effects		No	[145–147]
Viral-mediated APOE2 expression	Enhances APOE-connected neurodefensive effects	Mouse	Yes	[166, 169, 199]
A*β*12-28P, A*β*20-29 peptide, small-molecule inhibitors	Increases amyloid clearance	Mouse	No	[153–157]
Cyclosporine A	Decreases leakage of blood-derived toxic molecules in *APOE4*-carrying brain	Mouse	Yes	[179, 200]
